# Canagliflozin Attenuates PromarkerD Diabetic Kidney Disease Risk Prediction Scores

**DOI:** 10.3390/jcm12093247

**Published:** 2023-05-01

**Authors:** Kirsten E. Peters, Scott D. Bringans, Ronan S. O’Neill, Tasha S. C. Lumbantobing, James K. C. Lui, Timothy M. E. Davis, Michael K. Hansen, Richard J. Lipscombe

**Affiliations:** 1 Proteomics International, QEII Medical Centre, 6 Verdun Street, Nedlands, WA 6009, Australia; 2 Medical School, The University of Western Australia, Fremantle Hospital, Fremantle, WA 6959, Australia; 3 Janssen Research and Development, LLC, Spring House, PA 19477, USA

**Keywords:** type 2 diabetes, diabetic nephropathy, kidney decline, chronic kidney disease, biomarkers, risk prediction, prognosis

## Abstract

PromarkerD is a biomarker-based blood test that predicts kidney function decline in people with type 2 diabetes (T2D) who may otherwise be missed by current standard of care tests. This study examined the association between canagliflozin and change in PromarkerD score (Δ score) over a three-year period in T2D participants in the CANagliflozin cardioVascular Assessment Study (CANVAS). PromarkerD scores were measured at baseline and Year 3 in 2008 participants with preserved kidney function (baseline eGFR ≥60 mL/min/1.73 m^2^). Generalized estimating equations were used to assess the effect of canagliflozin versus placebo on PromarkerD scores. At baseline, the participants (mean age 62 years, 32% females) had a median PromarkerD score of 3.9%, with 67% of participants categorized as low risk, 14% as moderate risk, and 19% as high risk for kidney function decline. After accounting for the known acute drop in eGFR following canagliflozin initiation, there was a significant treatment-by-time interaction (*p* < 0.001), whereby participants on canagliflozin had decreased mean PromarkerD scores from baseline to Year 3 (Δ score: −1.0% [95% CI: −1.9%, −0.1%]; *p* = 0.039), while the scores of those on placebo increased over the three-year period (Δ score: 6.4% [4.9%, 7.8%]; *p <* 0.001). When stratified into PromarkerD risk categories, participants with high risk scores at baseline who were randomized to canagliflozin had significantly lower scores at Year 3 (Δ score: −5.6% [−8.6%, −2.5%]; *p* < 0.001), while those on placebo retained high scores (Δ score: 4.5% [0.3%, 8.8%]; *p* = 0.035). This post hoc analysis of data from CANVAS showed that canagliflozin significantly lowered PromarkerD risk scores, with the effect greatest in those T2D participants who were classified at study entry as at high risk of a subsequent decline in kidney function.

## 1. Introduction

Over 500 million (10%) adults globally were estimated to have diabetes in 2021, with a projected rise to over 780 million (12%) by 2045 [1]. The incidences of diabetes-related complications associated with financial stress, significantly reduced quality of life, and increased risk of death are also expected to rise [2]. One of these complications is end-stage kidney disease (ESKD), which constitutes around 7% of total US Medicare expenditures and has a mortality rate three times higher than that of cancer [3]. The primary cause of ESRD in most developed countries is diabetes-related chronic kidney disease (DKD); this is also the case in a growing number of developing countries, particularly those experiencing rapid increases in diabetes prevalence [2].

The current standard of care (SoC) for monitoring chronic kidney disease (CKD) relies primarily on serial measurement of the estimated glomerular filtration rate (eGFR) and urinary albumin–creatinine ratio (uACR). These tests provide metrics of kidney function at a specific point in time, offering limited insight into the trajectory ahead at an individual level due to both inter- and intra-patient variability [4,5]. To provide the best possible care for people with diabetes, with or without symptoms of kidney disease, a more accurate risk prediction tool for personalized preventative care is required.

PromarkerD is a validated, plasma-based biomarker test that can predict the onset and progression of kidney function decline in people with type 2 diabetes (T2D) [6,7,8]. In a recent study, PromarkerD was found to significantly outperform both eGFR and uACR in predicting kidney decline in community-based participants with T2D, correctly identifying 84% of participants with normal kidney function (eGFR ≥ 60 mL/min/1.73 m^2^ and uACR < 30 mg/g) who progressed to CKD (eGFR < 60 mL/min/1.73 m^2^) in the next four years, and who may otherwise have been missed [9]. In addition, PromarkerD classified 89% of participants known to be at risk of further kidney decline according to SoC, with elevated uACR (≥30 mg/g) and/or kidney impairment (eGFR < 60 mL/min/1.73 m^2^) [9]. With new blood glucose-lowering therapies, such as sodium-glucose cotransporter-2 inhibitors (SGLT2i), emerging as strong candidates for renoprotection [10], there is a need for identification of participants at highest risk of future CKD and, thus, likely to benefit the most from early initiation of these therapies. PromarkerD may have clinical utility in this respect.

The aim of the present study was, therefore, to examine whether the SGLT2i canagliflozin attenuated the PromarkerD risk score over a three-year follow-up period in participants with type 2 diabetes from the multinational CANagliflozin cardioVascular Assessment Study (CANVAS), a randomized placebo-controlled trial.

## 2. Materials and Methods

### 2.1. Participants

The present study was a post hoc analysis of data from the completed CANVAS program, which was a randomized controlled trial of canagliflozin versus placebo in people with type 2 diabetes at high risk for cardiovascular disease (ClinicalTrial.gov registration number NCT01032629). Details of the CANVAS study design and inclusion/exclusion criteria were previously reported [11]. A total of 2008 participants (*n* = 629 placebo arm, *n* = 1379 canagliflozin arm) from the modified intention-to-treat CANVAS population with preserved kidney function (baseline eGFR ≥ 60 mL/min/1.73 m^2^ with any uACR) and plasma samples available for analysis were included in the present study [11]. Urinary ACR was not used to classify the target population in this study. All demographic, biochemical, and clinical data were obtained from the CANVAS trial visits at the time of randomization (baseline visit) and at 156 weeks post-randomization (Year 3 visit). The key clinical variables included age, serum HDL-cholesterol, eGFR, and uACR. The CKD EPI equation was used for calculating eGFR [12].

### 2.2. PromarkerD Scores

PromarkerD scores were calculated at baseline and Year 3 using a previously defined algorithm that combines protein biomarker concentrations (apolipoprotein A-IV (apoA4), CD5 antigen-like (CD5L), such as insulin-like growth factor–binding protein 3 (IGFBP3)), with clinical data (age, serum HDL-cholesterol, eGFR) at each respective time point [7]. Protein biomarkers were measured using archived plasma samples stored at −80 °C via two antibody-based immunoassays, immunoaffinity mass spectrometry for baseline biomarker concentrations, and the PromarkerD CaptSure™ enzyme-linked immunosorbent assay (ELISA) (TGR Biosciences (an Abcam company), Perth, Australia) for Year 3 biomarker concentrations. Details of the immunoaffinity mass spectrometry and ELISA assays were previously described, including the agreement between methods [13,14]. In this sub-study, PromarkerD scores were predicted probabilities of incident CKD (eGFR <60 mL/min/1.73 m^2^ during four years of follow-up in those above this threshold at baseline), ranging from 0% to 100% and categorized as low, moderate or high risk, as determined using pre-specified cut-offs at 10% and 20% for optimal sensitivity and specificity [7]. Participants with scores <10% were categorized as ‘low’ risk, 10% to <20% as ‘moderate’ risk, and ≥20% as ‘high’ risk.

### 2.3. Statistical Analysis PromarkerD Scores

Baseline demographic and clinical characteristics are presented based on treatment allocation. All data are summarized as proportions, mean ± standard deviation (SD), geometric mean (SD range), or, in the case of variables which did not conform to a normal or log_e_-normal distribution (ln), median and interquartile range [IQR]. For independent samples, two-way comparisons for proportions were carried out via Chi-squared test, for normally distributed variables via Student’s *t*-test, and for non-normally distributed variables via Mann–Whitney *U*-test. Baseline PromarkerD scores were compared using treatment arm using unadjusted scores, as these were measured prior to treatment initiation. For the analysis of change in PromarkerD score, an adjustment to baseline values in participants on canagliflozin was made to account for the known transient acute drop in eGFR following treatment initiation. Adjusted PromarkerD scores were calculated using the Week 6 eGFR value, rather than the baseline eGFR in participants randomized to canagliflozin, keeping all other input variables from the baseline visit [15]. Generalized estimating equations were used to assess the effect of canagliflozin versus placebo on PromarkerD scores during the 3-year follow-up period, with effects also assessed via baseline unadjusted PromarkerD risk categories. Statistical analyses were performed in STATA (version 17.0; StataCorp. 2021) and SPSS Statistics Subscription (version 128.0.1.0 (142); SPSS Inc., Chicago, IL, USA). A two-tailed level of significance of *p <* 0.05 was used throughout.

## 3. Results

### 3.1. Baseline Participant Characteristics

The baseline clinical and demographic characteristics of the 2008 participants in the present CANVAS sub-study are presented in Table 1. The participants had a mean ± SD age of 61.7 ± 7.4 years, 32% were females, and their median [IQR] diabetes duration was 12.0 [8.0–17.0] years. In terms of kidney function, the mean eGFR was 82.3 ± 15.6 mL/min/1.73 m^2^ and the median uACR was 11.1 [6.3–30.9] mg/g. All participants at baseline had preserved kidney function (eGFR ≥ 60 mL/min/1.73 m^2^), with 21.4% being microalbuminuric (uACR 30–300 mg/g) and 4.2% being macroalbuminuric (uACR > 300 mg/g). The unadjusted median [IQR] PromarkerD score was 3.9% [0.7–14.5%], with 67% of participants categorized as low risk, 14% as moderate risk, and 19% as high risk for incident CKD. There was no statistically significant difference in participant characteristics or PromarkerD test scores via treatment allocation at baseline.

### 3.2. Effect of Canagliflozin on Change in PromarkerD Score

Adjusted baseline PromarkerD scores were calculated in participants on canagliflozin using the Week 6 eGFR value instead of the baseline value. As a result, 33 participants were excluded from subsequent analysis due to missing data. There was no significant difference in demographic or clinical characteristics between subjects excluded (*n* = 33) and included in the final analysis (*n* = 1975). As a result of the adjustment, a significant difference in mean baseline PromarkerD scores by treatment arm was observed, with higher scores found in participants on canagliflozin compared to those on placebo (18.1% [95% CI: 16.9%, 19.3%] versus 9.6% [8.6%, 10.6%]; *p <* 0.001). This difference is a result of the known acute drop in eGFR following canagliflozin initiation, which causes PromarkerD scores to increase artificially. There was no significant difference in PromarkerD scores between treatment arms at Year 3 (*p* = 0.29).

**Figure 1:**
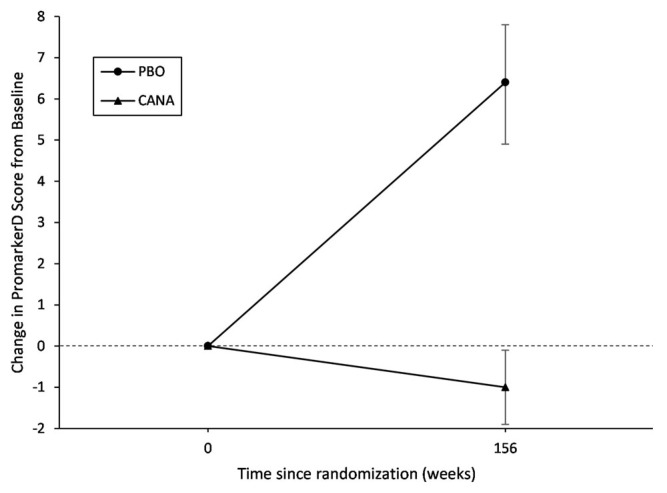
Change in PromarkerD scores during follow-up in 1975 participants from CANVAS trial.

**Figure 2:**
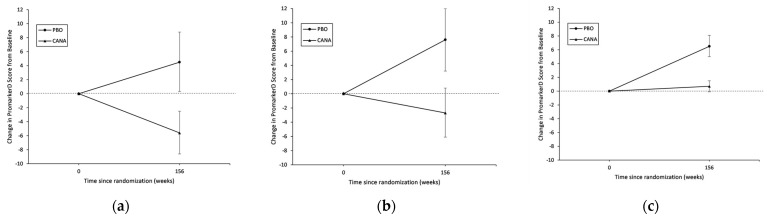
Change in PromarkerD scores during follow-up in 1975 participants from CANVAS trial by baseline PromarkerD risk category. (**a**) PromarkerD high-risk (*n* = 377); (**b**) PromarkerD moderate-risk (*n* = 281); (**c**) PromarkerD low-risk (*n* = 1317).

Across all 1975 participants, those on canagliflozin had significantly decreased mean PromarkerD scores from baseline to Year 3 (Δ score: −1.0% [95% CI: −1.9%, −0.1%]; *p* = 0.039), while those on placebo increased over the three-year period (Δ score: 6.4% [4.9%, 7.8%]; *p <* 0.001) (Table 2 and Figure 1). The mean change from baseline to Year 3 in PromarkerD scores between participants on placebo compared to canagliflozin was also significant (*p* < 0.001). The effect of canagliflozin on PromarkerD scores was greatest for participants classified in the high-risk category, where scores decreased by 5.6% (95% CI: −8.6%, −2.5%), while scores in those on placebo increased by 4.5% (0.3%, 8.8%). Canagliflozin was also associated with decreased PromarkerD scores for those in the moderate-risk category (Δ score: −2.7% [−6.1%, 0.8%]), although this did not reach statistical significance. PromarkerD scores remained stable for participants in the low-risk category on canagliflozin (Δ score: 0.7% [−0.1%, 1.5%]) (Table 2 and Figure 2).

## 4. Discussion

Previous studies demonstrated that higher PromarkerD scores are significantly predictive of incident CKD in the next four years in community-based and clinical trial participants with type 2 diabetes [7,8]. Moreover, moderate- and high-risk PromarkerD scores are increasingly prognostic for outcomes, allowing a more informed approach to management of those at highest risk of developing CKD. In the present study, canagliflozin significantly decreased mean PromarkerD scores compared to placebo over three years, with the effect greatest for those classified using PromarkerD as at high risk of a subsequent decline in kidney function at the outset. In contrast, PromarkerD scores for participants on placebo significantly increased during the three years. It follows that a decrease in PromarkerD scores is associated with a lower risk of future CKD, as well as longer term beneficial kidney and cardiovascular disease outcomes.

It is well established that canagliflozin induces a reversible acute drop in eGFR following treatment initiation, followed by stabilization of longer-term eGFR trajectories [16,17]. As eGFR is a key component of calculation of the PromarkerD score, any analysis of change in scores post-treatment would be problematic unless this initial drop in eGFR is taken into consideration. As suggested in prior studies, the first on-treatment eGFR (Week 6) can be used as the baseline value to account for this initial drop [15]. This initial fall in eGFR, together with normal decline in eGFR with the increasing age of participants over time, was expected to result in increased PromarkerD scores. To further investigate this premise, the change in each individual component of the score was assessed over the three-year period to rule out the possibility that increases in scores were due only to changes in clinical factors, while confirming that the PromarkerD biomarkers were also changing. These analyses showed that biomarker changes did contribute to changes in risk scores. The significant decrease in PromarkerD scores in participants on canagliflozin suggests that treatment may affect one or more of the pathophysiological pathways captured using the individual biomarkers. Indeed, there is a growing body of evidence to support the utility of these biomarkers in the prediction of CKD progression [18,19,20,21].

Despite the contemporary recommended focus on tight glycaemic, lipid, and blood pressure control, a significant number of people with type 2 diabetes still develop kidney disease, which, in turn, is associated with an increased risk of cardiovascular events. It is imperative that CKD progression is reduced in type 2 diabetes: PromarkerD allows earlier identification of those at highest risk of adverse outcomes. Tests such as PromarkerD would support cost-effective individualised treatment. In addition, serial PromarkerD testing seems useful as a way of monitoring the individual patient response to management changes. A recent survey of 400 physicians evaluated the importance of PromarkerD for clinical decision making [22]. This survey showed that, compared with no PromarkerD availability, a high-risk PromarkerD result was significantly associated with increased renal risk factor monitoring, SGLT2i prescription, and lisinopril dose, while a low-risk PromarkerD result was significantly associated with decreased risk factor monitoring and reduced SGLT2i prescription [22].

The major strengths of the present study are its longitudinal design and inclusion of over 1900 well-characterized people with type 2 diabetes from the CANVAS program. CANVAS is a large multi-center clinical trial of canagliflozin, with plasma stored at each trial visit for additional testing. This approach allowed PromarkerD scores to be measured before and after therapy initiation, and over a time horizon relevant to people with type 2 diabetes and clinicians managing diabetes and DKD risk. The present study also had limitations. The prognostic utility of change in PromarkerD scores for predicting future kidney outcomes was not considered; the effect of other clinical and therapeutic changes on PromarkerD scores over time was also excluded from the study’s scope. The generalizability of the results to other racial and ethnic groups is limited as the participants were mostly Caucasian (82%).

The present post hoc analysis of data from the CANVAS program provides the first evidence that the SGLT2i canagliflozin can attenuate PromarkerD diabetic kidney disease risk prediction scores over a three-year period. This study extends previous work that showed the prognostic utility of PromarkerD for predicting kidney outcomes in type 2 diabetes, and confirms that the test identifies individuals at highest risk who would benefit the most from early intervention, as demonstrated via improvement in PromarkerD scores and the associated kidney risk profiles.

## Figures and Tables

**Table 1:** Baseline demographic and clinical characteristics of 2008 CANVAS participants used for PromarkerD analysis via treatment arm.

Characteristic	PBO	CANA	Total
Number of samples (%)	629 (31.3%)	1379 (68.7%)	2008
Age (years)	61.3 ± 7.2	61.8 ± 7.4	61.7 ± 7.4
Female sex, *n* (%)	200 (31.8)	432 (31.3)	632 (31.5)
BMI (kg/m^2^)	32.2 ± 5.7	32.5 ± 6.1	32.4 ± 5.9
Diabetes duration (years) *	12.0 [8.0–16.6]	12.1 [8.0–17.1]	12.0 [8.0–17.0]
Fasting plasma glucose (mmol/L) *	9.0 [7.5–10.9]	9.1 [7.6–11.0]	9.1 [7.6–10.9]
HbA_1c_ (%) *	8.0 [7.5–8.8]	8.0 [7.5–8.7]	8.0 [7.5–8.7]
Serum total cholesterol (mmol/L)	4.3 ± 1.2	4.3 ± 1.1	4.3 ± 1.1
Serum HDL-cholesterol (mmol/L)	1.21 ± 0.31	1.19 ± 0.32	1.20 ± 0.32
Serum triglycerides (mmol/L) ^†^	1.7 (1.0–2.8)	1.7 (1.0–2.8)	1.7 (1.0–2.8)
Systolic blood pressure (mmHg)	136 ± 16	136 ± 15	136 ± 15
Diastolic blood pressure (mmHg)	78 ± 9	78 ± 9	78 ± 9
Diuretic use, *n* (%)	281 (44.7)	593 (43.0)	874 (43.5)
History of heart failure, *n* (%)	81 (12.9)	157 (11.4)	238 (11.9)
Urine albumin to creatinine ratio (mg/g) *	10.9 [6.3–32.1]	11.1 [6.3–30.0]	11.1 [6.3–30.9]
eGFR (mL/min/1.73 m^2^)	82.2 ± 16.0	82.4 ± 15.5	82.3 ± 15.6
PromarkerD score (%) *^,¥^	3.7 [0.7–14.3]	3.7 [0.7–14.3]	3.9 [0.7–14.5]
PromarkerD risk category, *n* (%) ^¥^			
Low	421 (66.9)	919 (66.6)	1340 (66.7)
Moderate	99 (15.7)	188 (13.6)	287 (14.3)
High	109 (17.3)	272 (19.7)	381 (19.0)

All values are mean ± SD (standard deviation) unless labeled otherwise; * Median (IQR—interquartile range); ^†^ Geometric Mean (SD range); ^¥^ Baseline PromarkerD scores in this table are unadjusted as measured prior to treatment initiation. Abbreviations: PBO, placebo; CANA, canagliflozin; BMI, body mass index; eGFR, estimated glomerular filtration rate based on CKD Epidemiology Collaboration equation.

**Table 2:** Change in PromarkerD scores during follow-up in 1975 participants from CANVAS trial.

				Differences in the Means *		
	N	Mean	95% CI	Δ PromarkerD	95% CI	*p*-Value
**All Participants (*n* = 1975)**						
Placebo						
Baseline	629	9.6	8.6, 10.6	ref		
Year 3	629	16.0	14.3, 17.7	6.4	4.9, 7.8	<0.001
Canagliflozin						
Baseline	1346	18.1	16.9, 19.3	ref		
Year 3	1346	17.1	15.9, 18.3	−1.0	−1.9, −0.1	0.039
**PromarkerD High-Risk (*n* = 377)**						
Placebo						
Baseline	109	33.2	31.2, 35.2	ref		
Year 3	109	37.7	33.4, 42.1	4.5	0.3, 8.8	0.035
Canagliflozin						
Baseline	268	48.5	45.8, 51.1	ref		
Year 3	268	42.9	39.6, 46.2	−5.6	−8.6, −2.5	<0.001
**PromarkerD Moderate-Risk (*n* = 281)**						
Placebo						
Baseline	99	14.2	13.7, 14.8	ref		
Year 3	99	21.8	17.4, 26.3	7.6	3.2, 12.0	<0.001
Canagliflozin						
Baseline	182	27.6	25.1, 30.1	ref		
Year 3	182	24.9	21.9, 28.0	−2.7	−6.1, 0.8	0.14
**PromarkerD Low-Risk (*n* = 1317)**						
Placebo						
Baseline	421	2.4	2.2, 2.7	ref		
Year 3	421	9.0	7.4, 10.6	6.5	5.0, 8.1	<0.001
Canagliflozin						
Baseline	896	7.1	6.4, 7.9	ref		
Year 3	896	7.8	7.0, 8.7	0.7	−0.1, 1.5	0.091

* Estimation from GEE models for repeated measures assuming a log-normal distribution and an interaction term between time period and treatment and estimation of robust standard errors.

## Data Availability

Data from the overall CANVAS Program are available in the public domain via the Yale University Open Data Access Project (YODA; http://yoda.yale.edu/ (accessed on 15 December 2022)). The data specific to the present study are available from the authors upon reasonable request.

## References

[1] Sun H., Saeedi P., Karuranga S., Pinkepank M., Ogurtsova K., Duncan B.B., Stein C., Basit A., Chan J.C.N., Mbanya J.C. (2022). IDF Diabetes Atlas: Global, regional and country-level diabetes prevalence estimates for 2021 and projections for 2045. Diabetes Res. Clin. Pract..

[2] Koye D.N., Magliano D.J., Reid C.M., Jepson C., Feldman H.I., Herman W.H., Shaw J.E. (2018). Risk of Progression of Nonalbuminuric CKD to End-Stage Kidney Disease in People With Diabetes: The CRIC (Chronic Renal Insufficiency Cohort) Study. Am. J. Kidney Dis..

[3] National Institute of Diabetes and Digestive and Kidney Diseases United States Renal Data System 2022 Annual Data Report. https://usrds-adr.niddk.nih.gov/2022.

[4] Kidney Disease: Improving Global Outcomes Chronic Kidney Disease Guideline Development Work Group Members (2013). KDIGO 2012 Clinical practice guideline for the evaluation and management of chronic kidney disease. Kidney Int. Suppl..

[5] Kerschbaum J., Rudnicki M., Dzien A., Dzien-Bischinger C., Winner H., Heerspink H.L., Rosivall L., Wiecek A., Mark P.B., Eder S. (2020). Intra-individual variability of eGFR trajectories in early diabetic kidney disease and lack of performance of prognostic biomarkers. Sci. Rep..

[6] Peters K.E., Davis W.A., Ito J., Winfield K., Stoll T., Bringans S.D., Lipscombe R.J., Davis T.M.E. (2017). Identification of Novel Circulating Biomarkers Predicting Rapid Decline in Renal Function in Type 2 Diabetes: The Fremantle Diabetes Study Phase II. Diabetes Care.

[7] Peters K.E., Davis W.A., Ito J., Bringans S.D., Lipscombe R.J., Davis T.M.E. (2019). Validation of a protein biomarker test for predicting renal decline in type 2 diabetes: The Fremantle Diabetes Study Phase II. J. Diabetes Complicat..

[8] Peters K.E., Xu J., Bringans S.D., Davis W.A., Davis T.M.E., Hansen M.K., Lipscombe R.J. (2020). PromarkerD Predicts Renal Function Decline in Type 2 Diabetes in the Canagliflozin Cardiovascular Assessment Study (CANVAS). J. Clin. Med..

[9] Peters K., Bringans S., Davis W., Lipscombe R., Davis T. A Comparison of PromarkerD to Standard-of-Care Tests for Predicting Renal Decline in Type 2 Diabetes. Proceedings of the American Society of Nephrology Kidney Week.

[10] de Boer I.H., Caramori M.L., Chan J.C.N., Heerspink H.J.L., Hurst C., Khunti K., Liew A., Michos E.D., Navaneethan S.D., Olowu W.A. (2020). Executive summary of the 2020 KDIGO Diabetes Management in CKD Guideline: Evidence-based advances in monitoring and treatment. Kidney Int..

[11] Neal B., Perkovic V., Mahaffey K.W., de Zeeuw D., Fulcher G., Erondu N., Shaw W., Law G., Desai M., Matthews D.R. (2017). Canagliflozin and Cardiovascular and Renal Events in Type 2 Diabetes. N. Engl. J. Med..

[12] Levey A.S., Stevens L.A., Schmid C.H., Zhang Y.L., Castro A.F., Feldman H.I., Kusek J.W., Eggers P., Van Lente F., Greene T. (2009). A new equation to estimate glomerular filtration rate. Ann. Intern. Med..

[13] Bringans S., Ito J., Casey T., Thomas S., Peters K., Crossett B., Coleman O., Ebhardt H.A., Pennington S.R., Lipscombe R. (2020). A robust multiplex immunoaffinity mass spectrometry assay (PromarkerD) for clinical prediction of diabetic kidney disease. Clin. Proteom..

[14] Bringans S., Peters K., Casey T., Ito J., Lipscombe R. (2020). The New and the Old: Platform Cross-Validation of Immunoaffinity MASS Spectrometry versus ELISA for PromarkerD, a Predictive Test for Diabetic Kidney Disease. Proteomes.

[15] Oshima M., Neal B., Toyama T., Ohkuma T., Li Q., de Zeeuw D., Heerspink H.J.L., Mahaffey K.W., Fulcher G., Canovatchel W. (2020). Different eGFR Decline Thresholds and Renal Effects of Canagliflozin: Data from the CANVAS Program. J. Am. Soc. Nephrol..

[16] Perkovic V., de Zeeuw D., Mahaffey K.W., Fulcher G., Erondu N., Shaw W., Barrett T.D., Weidner-Wells M., Deng H., Matthews D.R. (2018). Canagliflozin and renal outcomes in type 2 diabetes: Results from the CANVAS Program randomised clinical trials. Lancet Diabetes Endocrinol..

[17] Perkovic V., Jardine M.J., Neal B., Bompoint S., Heerspink H.J.L., Charytan D.M., Edwards R., Agarwal R., Bakris G., Bull S. (2019). Canagliflozin and Renal Outcomes in Type 2 Diabetes and Nephropathy. N. Engl. J. Med..

[18] Kronenberg F. (2017). Apolipoprotein L1 and apolipoprotein A-IV and their association with kidney function. Curr. Opin. Lipidol..

[19] Yang H., Luo Y., Lai X. (2022). The comprehensive role of apoptosis inhibitor of macrophage(AIM) in pathological conditions. Clin. Exp. Immunol..

[20] Miyazaki T., Yamazaki T., Sugisawa R., Gershwin M.E., Arai S. (2018). AIM associated with the IgM pentamer: Attackers on stand-by at aircraft carrier. Cell. Mol. Immunol..

[21] Wang S., Chi K., Wu D., Hong Q. (2021). Insulin-Like Growth Factor Binding Proteins in Kidney Disease. Front. Pharmacol..

[22] Fusfeld L., Murphy J.T., Yoon Y., Kam L.Y., Peters K.E., Lin Tan P., Shanik M., Turchin A. (2022). Evaluation of the clinical utility of the PromarkerD in-vitro test in predicting diabetic kidney disease and rapid renal decline through a conjoint analysis. PLoS ONE.

